# Optimizing malaria vector control in the Greater Mekong Subregion: a systematic review and mathematical modelling study to identify desirable intervention characteristics

**DOI:** 10.1186/s13071-024-06234-4

**Published:** 2024-03-29

**Authors:** Yuqian Wang, Nakul Chitnis, Emma L. Fairbanks

**Affiliations:** 1 Department of Epidemiology and Public Health, Swiss Tropical and Public Health Institute, Kreuzstrasse 2, Allschwill, 4123 Basel, Switzerland; 2 University of Basel, Petersplatz 1, 4001 Basel, Switzerland

**Keywords:** Malaria, *Anopheles*, Bionomics, Vectorial capacity, Human blood index, Parity, Sac, Gonotrophic cycle, Global sensitivity analysis, Partial rank correlation coefficients, Sobol’s method

## Abstract

**Background:**

In the Greater Mekong Subregion (GMS), new vector-control tools are needed to target mosquitoes that bite outside during the daytime and night-time to advance malaria elimination.

**Methods:**

We conducted systematic literature searches to generate a bionomic dataset of the main malaria vectors in the GMS, including human blood index (HBI), parity proportion, sac proportion (proportion with uncontracted ovary sacs, indicating the amount of time until they returned to host seeking after oviposition) and the resting period duration. We then performed global sensitivity analyses to assess the influence of bionomics and intervention characteristics on vectorial capacity.

**Results:**

Our review showed that *Anopheles minimus*, *An. sinensis*, *An. maculatus* and *An. sundaicus* display opportunistic blood-feeding behaviour, while *An. dirus* is more anthropophilic. Multivariate regression analysis indicated that environmental, climatic and sampling factors influence the proportion of parous mosquitoes, and resting duration varies seasonally. Sensitivity analysis highlighted HBI and parity proportion as the most influential bionomic parameters, followed by resting duration. Killing before feeding is always a desirable characteristic across all settings in the GMS. Disarming is also a desirable characteristic in settings with a low HBI. Repelling is only an effective strategy in settings with a low HBI and low parity proportion. Killing after feeding is only a desirable characteristic if the HBI and parity proportions in the setting are high.

**Conclusions:**

Although in general adopting tools that kill before feeding would have the largest community-level effect on reducing outdoor transmission, other modes of action can be effective. Current tools in development which target outdoor biting mosquitoes should be implemented in different settings dependent on their characteristics.

**Graphical Abstract:**

**Figure:**
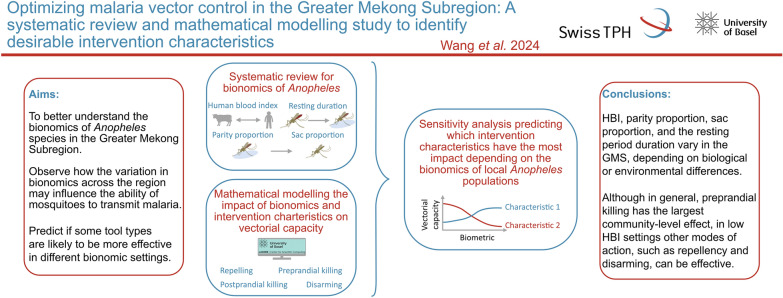

**Supplementary Information:**

The online version contains supplementary material available at 10.1186/s13071-024-06234-4.

## Background

Malaria is an infectious disease transmitted to humans and other animals through the infectious female *Anopheles* mosquito. It is caused by *Plasmodium* parasites, namely *P. falciparum*, *P. vivax*, *P. knowlesi*, *P. malariae*, *P. ovale curtisi* and *P. ovale wallikeri* [1]. *P. falciparum* and *P. vivax* are the main global health threat [2]. *P. falciparum* malaria can cause severe malaria, and if not treated properly, can lead to mortality [3]. The wide use of vector control tools, including insecticide-treated nets (ITNs) and indoor residual spraying (IRS), along with available treatments such as artemisinin-based combination therapy (ACT), have dramatically decreased malaria disease burden [4]. However, malaria remains a global health challenge, and caused an estimated 247 million cases and 619,000 deaths worldwide in 2021 [2].

The Greater Mekong Subregion (GMS), located in the World Health Organization (WHO) Southeast Asia region, consists of six countries, including Cambodia, China (Yunnan province), Lao PDR, Myanmar, Thailand and Vietnam. In GMS, *P. vivax*, *P. falciparum* and mixed malaria are the prevailing species of malaria [5]. As *P. falciparum* cases have decreased, *P. vivax* has become the main parasite in this region [2]. Among the numerous vector species reported in GMS, *An. minimus* Theobald 1901 and *An. dirus* Peyton and Harrison 1979 are primary vector species. Additionally, other *Anopheles* species, including *An. sundaicus* Rodenwalt 1926, *An. sinensis* Wiedemann 1925 and *An. maculatus* Theobald 1901 are also able to transmit malaria parasites [6].

However, insecticide resistance for pyrethroids (including permethrin and deltamethrin) and organochlorines (DDT) has been found in multiple malaria vectors in the region [7–9], threatening the efficacy of malaria vector control. Additionally, multi-drug-resistant *P. falciparum* malaria parasites have developed in this region and migrated to other countries [10], reducing the effectiveness of many therapies. Therefore, GMS remains a focal point for malaria elimination.

Most of the malaria-endemic regions within the GMS are located in the forest, forest fringes and around international borders [11]. In the forest, mosquitoes have a tendency to bite outdoors as well as during the daytime, reducing the effect of current vector tools [12]. Therefore, towards the elimination of malaria in GMS, new vector tools targeting mosquitoes that bite outside, bite during the daytime and are resistant to common insecticide are urgently needed.

Mathematical modelling plays an essential role in tool selection. To assess the influence of model parameters on output, sensitivity analysis is commonly used [13]. Over the years, vector control tool selection has been supported by sensitivity analysis of vectorial capacity [14]. Vectorial capacity, referring to the capacity of mosquitoes to transmit malaria parasites, is defined as the average number of potentially infectious bites on all hosts from mosquitoes infected by one initial host in one unit of time, in the event that every female mosquito becomes infectious after biting a malaria-infected host [15]. Vectorial capacity helps predict the community-level effect of using a new vector tool on *Plasmodium* transmission [16–18].

Several bionomic parameters are included in different mathematical models for estimating vectorial capacity [14, 19, 20], primarily consisting of daily survival and mortality probabilities, the ratio of mosquitoes compared with humans and the biting rate [21]. In our analysis we consider a vectorial capacity model derived from a discrete-time entomological model of the *Anopheles* feeding cycle, in Chitnis et al. [22] and Briët et al. [16]. This model was chosen since it looks at the effects of interventions at different stages during each mosquito feeding cycle. The parameter values of the model are derived from several measurable bionomics parameters: human blood index, parity proportion, sac proportion, resting period duration and other standard parameters such as the probability that a mosquito survives or dies at each stage of the feeding cycle. Human blood index (HBI), the proportion of mosquitoes that have fed on humans out of all blood-feeding mosquitoes analysed, informs the host preference of mosquitoes and the availability of local animals to mosquitoes. In addition, HBI and mosquito feeding frequency determine the human-biting habit of mosquitoes. Parity proportion, often referred to as the parity rate, is the proportion of mosquitoes that have previously laid eggs. This relates the mortality of mosquitoes, the life span of mosquitoes, adult mosquito emergence rate and the duration of a gonotrophic cycle to each other. Therefore, variations in the parity proportion reflect many aspects of mosquito population dynamics [23]. Sac proportion, often referred to as the sac rate, is the proportion of mosquitoes with uncontracted ovary sacs, indicating they returned to host-seeking within a day of oviposition [24]. Along with the resting period duration of a mosquito, which is the duration required for blood digestion and ovaries maturation, the sac proportion can be used to estimate the average duration of the mosquito feeding cycle [22, 24]. Here, we assume the time to develop eggs is much longer than the time to find an oviposition site, and therefore mosquitoes leave their resting location and oviposit the same day.

Systemic literature reviews can help to parameterize mathematical models. A global bionomics database for the main malaria vectors was established in 2010, containing field data from 1985 to 2010 [25]. However, the parameters may vary after a decade. In addition, this database did not collect data on sac proportion. More recently, Orsborne et al. [26] performed a systematic review of the HBI for three major African malaria vectors. To identify the desirable characteristics of new mosquito control tools in the GMS, an updated dataset of the local mosquito entomological parameters is needed.

In this paper, we generate a dataset of these mosquito-related parameters from a systematic search. Then we conduct global sensitivity analysis on the Chitnis et al. [22] model to understand how these parameters affect the ability of mosquitoes to transmit malaria. Furthermore, sensitivity analysis was also used to identify key performance properties of vector control tools depending on the bionomics of the local mosquito populations in the GMS.

## Methods

### Systematic literature search

**Table 1:** Search terms used for the systematic searches

Parameter	Pubmed search term
Human blood index	“Anopheles” AND (“minimus” OR “dirus” OR “maculatus” OR “sinensis” OR “sundaicus”) AND (“Human blood index” OR “HBI” OR “host preference” OR “trophic preference” OR “blood meal preference” OR “blood host preference” OR “blood meal” OR “blood meal analysis” OR “blood-meal analysis” OR “blood meal source” OR “host blood” OR “host blood meal” OR “blood meal identification” OR “anthropophilic index” OR “human blood fed”)
Parity proportion, sac proportion and resting period duration	“Anopheles” AND (“minimus” OR “dirus” OR “maculatus” OR “sinensis” OR “sundaicus”) AND (“parity” OR “parous” OR “multiparous” OR “bionomics” OR “gonotrophic cycle” OR “oocyst” OR “entomological” OR “entomologic” OR “vectorial capacity” OR “ecology” OR “ecological” OR “survival” OR “oviposition” OR “dissection” OR “mortality” OR “sporozoite” OR “sac”)

**Table 2:** Inclusion and exclusion criteria for the systematic searches

Inclusion criteria	Exclusion criteria
Reported human blood index (using blood meal analysis), parity proportion, sac proportion or resting period duration	Reported human blood index using other measurements (e.g. anthropophilic index) or without reporting human blood index, parity proportion, sac proportion or resting period duration
Reported bionomics for *An. dirus* complex, *An. minimus* complex, *An. sinensis*, *An. sundaicus* complex and *An. maculatus* group	Reported bionomics of other mosquito species or did not specify mosquito species
Original report	Review or repeat report
All languages	

#### Search strategy

The search terms (Table 1) and inclusion and exclusion criteria (Table 2) were utilised for the systematic search. Original studies reporting the HBI, parity proportion, sac proportion or resting period duration of *An. dirus* complex, *An. minimus* complex, *An. sinensis* complex, *An. sundaicus* complex or *An. maculatus* group were included. The Maculatus group, formerly known as a species complex, is currently considered as supercomplexes with subordinate complexes [27]. The publications without the bionomics of at least one of these five species complex and repeat reports were excluded.

#### Data extraction

Bionomic data were extracted from the eligible papers. Data describing other variables that could influence the bionomics were also collected, including species (complex), season (e.g. rainy, dry), trapping location (e.g. indoor, outdoor) and trapping method. For parity proportion, the concurrent use of insecticides for control purposes was also recorded.

The online resources were searched for variables not described in the article, including season and current usage of the vector control tool. If the season was not given, the rainy and dry seasons were calculated by searching for the regular rainy and dry seasons in the particular area [28]. Studies containing two seasons would be classified into the both category. Additionally, extreme situations such as exceptionally low or no rainfall in the rainy season would be classified into the both category. References in eligible articles, World Health Organization malaria reports [29, 30] and relative vector control publications [31–34] were read to assess the usage status of vector control.

Geo-position information including country, study location, area type, latitude and longitude was recorded for mapping the distribution of data points. In the absence of coordinates from the publication, online gazetteers such as Google Maps and Google Earth were used to determine the coordinate for the given study site, consistent with the Malaria Atlas Project MAP database [28].

### Statistical analysis

#### Descriptive statistics

For HBI, parity proportion and sac proportion, the mean and the 95% confidence interval (CI) were calculated, weighted by the total number of mosquitoes samples analysed. Data points with less than ten mosquito samples were excluded. For the resting period duration, the range is given. The data points were mapped using latitude and longitude coordinates referenced to the World Geodetic Reference System 1984 (WGS 84) ellipsoid to see the geographical distribution [35, 36].

#### Inferential statistics

For the generated bionomic datasets that contain a sample size of five or more for each species complex, the difference among species complexes was compared using a Kruskal–Wallis test. Moreover, the post hoc Dunn’s test was conducted for multiple pairwise comparisons. Here, Bonferroni adjusted *P*-values were used to account for multiple statistical tests being performed on a single dataset. A *P*-value less than 0.05 was considered significant.

Univariate and multivariate logistic regressions were carried out for the dependent variable: parity proportion, weighted by the total number of analysed mosquitoes. The independent variables were selected on the basis of factors known to affect parity proportion: species (complex), trapping location, trapping method, vector control tool usage status, season, climate zone and land use class.

Geographical variables, including climate zone and land use class, were extracted for the coordinates of each data point. The Koppen–Geiger climate classification map for the present day (1980–2016) [37] was used to categorize the climate into three types: tropical, temperate and cold. Additionally, the Terra and Aqua combined Moderate Resolution Imaging Spectroradiometer (MODIS) Land Cover Type (MCD12Q1) data product (2001–2019) [38] was used to categorize the land cover type following Food and Agriculture Organization Land Cover Classification System land use class (LCCS2). The land cover type was grouped into five categories: forests (open forests and dense forests), croplands, forest/cropland mosaics, urban and build-up lands and nature herbaceous. In addition, considering the impact of urbanization and deforestation on land use type, data points before 1990 were reviewed and updated.

Statistical analysis was performed using R version 4.1.2 [39] and RStudio [40].

### Modelling vectorial capacity

#### Entomology model

**Fig. 1:**
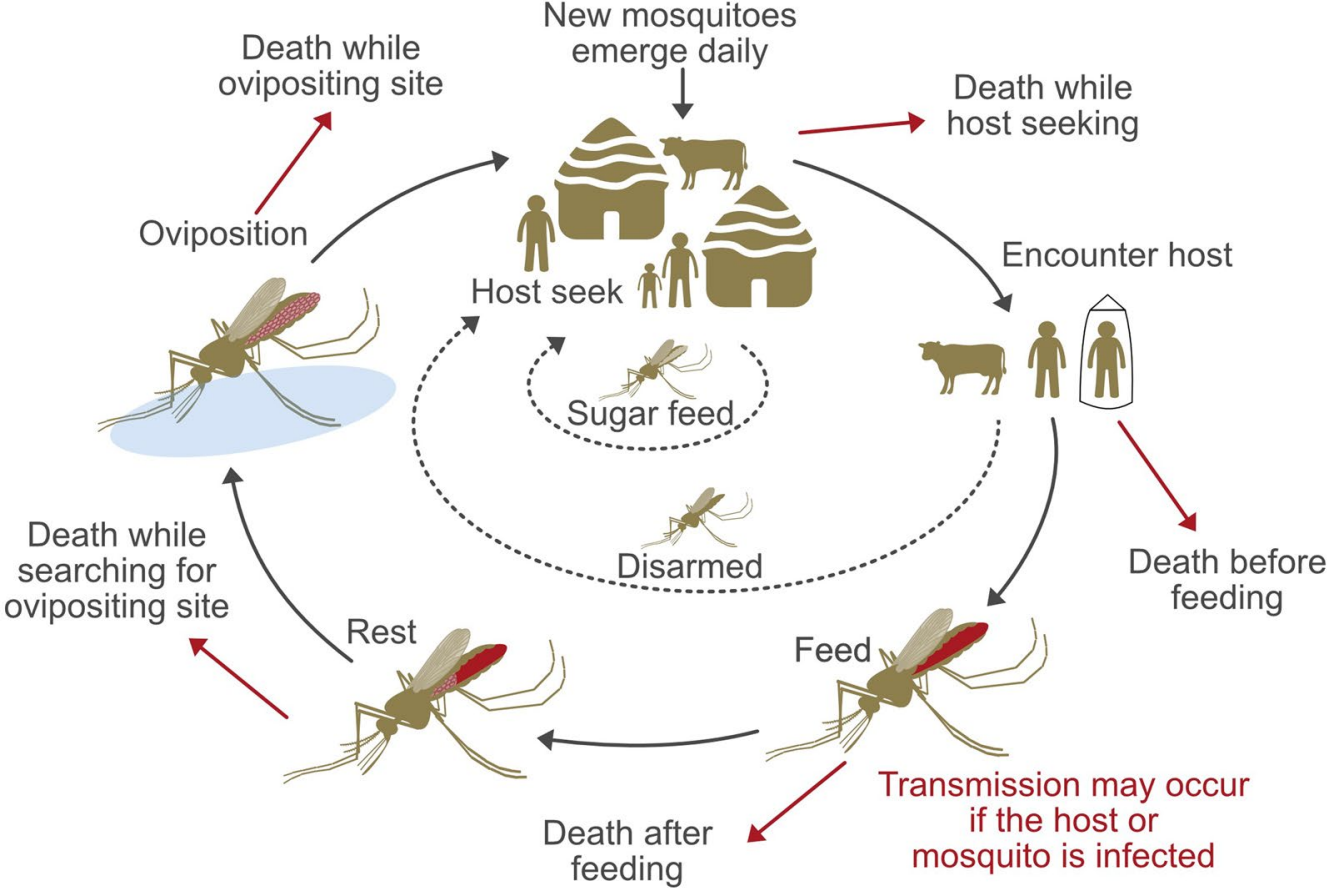
*Anopheles* feeding cycle model from [18]. Repelling reduces the host-encountering rate for protected hosts. Preprandial killing increases death before feeding (no parasite transmission). Disarmed mosquitoes do not bite any host for the remaining duration of the feeding cycle. Postprandial killing increases death after feeding (possible parasite transmission)

Our model framework uses a discrete-time entomological model of the *Anopheles* feeding cycle [22]. The feeding cycle in this model consists of five states: host-seeking, host-encountering, biting, resting and ovipositing (Fig. 1). Vectorial capacity was previously derived from this entomological model [22]. We used the vectorial capacity of mosquitoes to measure the potential for *Plasmodium* transmission, which is affected by local bionomics and vector tools. To compare the impact of the different bionomic parameters and intervention parameters, we used the relative change of vectorial capacity versus baseline vectorial capacity as our model output, since it is not affected by the environmental larval carrying capacity [17]. Baseline vectorial capacity was calculated using the default value of the parameters in Table 3.

#### Intervention model

To simulate the impact of the intervention characteristics, we integrated four modes of action parameters as possible effects of the novel vector tool into this model: the repelling effect, preprandial killing effect (killing before blood-feeding), disarming effect (biting inhibited for the duration of the feeding cycle remaining) and postprandial killing effect (killing after blood-feeding). Vector control tools which target host-seeking mosquitoes usually have one or more of these properties. For example, untreated bed nets repel mosquitoes and insecticide-treated bed nets aim to kill before feeding, however, as nets age or mosquitoes develop resistance, more mosquitoes may be disarmed or repelled instead [41]. Novel interventions, such as transfluthrin emulators (often called spatial repellents), kill, disarm and repel mosquitoes, depending on the transfluthrin concentration that the mosquito is exposed to [18, 42].

We specified three types of real hosts: unprotected malaria hosts (hosts without the tool), protected malaria host (hosts with the tool) and non-malaria host (such as cattle). A detailed description of model parameters and equations can be found in the Additional file 1. The number of protected hosts is given by multiplying the total number of malaria hosts with the intervention coverage level. Repelling, disarming and preprandial killing effect could contribute to the reduction of biting. The reduction in biting effect was incorporated into the model by decreasing the host availability rate of the protected host. The postprandial killing effect was modelled by reducing the probability of mosquitoes finding a resting place after feeding a protected host [17]. To include preprandial killing and disarming we include dummy hosts (with one dummy host per protected host). Dummy hosts are not real hosts, including these in the model allows for these mosquitoes to be removed from the system for the remainder of the feeding cycle. Vectors which interact with preprandial killing dummy hosts are killed. Disarming dummy hosts simulate the period where the mosquito remains disarmed before returning to host-seeking.

### Sensitivity analysis

#### Sensitivity analysis of the entomology model

Two global sensitivity analyses were performed to test how sensitive the model output is to the bionomic parameters. Using the relative change of vectorial capacity as our model output, we evaluated four bionomic parameters in the sensitivity analysis: human blood index, parity proportion, sac proportion and resting period duration. An overview of model parameters is presented in Table 3.

To explore the relationship between the parameters and output, we used a sampling-based sensitivity analysis method, Latin hypercube sampling – Partial rank correlation coefficients (LHS-PRCC). This approach can be applied in the case of nonlinear but monotonic relationships between model output and each model parameter [43]. Firstly, a Monte Carlo approach, Latin hypercube sampling (LHS), was used to generate samples. A total of 500 random parameter sets were generated from uniform distributions on the basis of the ranges listed in Table 3. Secondly, for each parameter set, the model output was obtained. Partial rank correlation coefficients (PRCC) between each parameter and output were then calculated. Moreover, 50 replicated LHS-PRCC were performed to compute the means and 95% confidence intervals of the PRCC. Significance tests were conducted to assess whether a PRCC significantly differed from zero at a 95% confidence level, considering the Bonferroni multiple test correction [43]. A positive PRCC indicates the positive correlation between the input parameter and the output, while a negative PRCC indicates the negative correlation between the input parameter and model output. PRCC close to 1 or −1 means the parameter is very influential to the model output [13].

To evaluate the effect of each parameter and the interactions of the parameters, we used a variance-based method, Sobol’s method [44]. Firstly, Sobol’s quasi-random numbers [45, 46] were adopted to generate samples. A total of 6000 parameter sets were generated from uniform distributions on the basis of each parameter range. For each parameter set, the output was calculated. Secondly, the first order Sobol’s indices, which measure the main effect of each parameter, were derived with the Saltelli estimator [47]. The total order Sobol’s indices, which measure the total effect of each parameter, were assessed with the Jansen estimators [48], showing good accuracy and efficiency [49]. Furthermore, 50 bootstrap replicas were used to generate the 95% confidence intervals. Lastly, the first order and total order Sobol’s indices of a dummy parameter were computed to determine the error from numerical approximation and identify the significant parameters [49].

#### Sensitivity analysis of the intervention model

To understand how sensitive the relative reduction of vectorial capacity was to the intervention parameters in different bionomic settings, we performed two global sensitivity analyses. We evaluated four intervention parameters in the sensitivity analysis: the repelling effect, preprandial killing effect, disarming effect and postprandial killing effect. The intervention parameters were derived from semi-field or field experiments, as presented in Table 3.

LHS-PRCC and Sobol’s method were conducted, considering three intervention coverage levels: 10%, 30% and 50%. When examining the sensitivity index in the variation range of one bionomic parameter, the other bionomic parameters were fixed to the mean value.

Sensitivity analysis was performed using R version 4.1.0 [39] and RStudio [40]. For LHS-PRCC, Latin hypercube samples were obtained with the LHS package [50], and the PRCC results were obtained using the sensitivity package [51]. The Sobol’s quasi-random numbers and the Sobol’s indices were obtained with the sensobol package [49].

**Table 3:** Parameter definition, default value and range of vectorial capacity model and intervention model. For the bionomic parameters the default value is the weighted mean across species and the range is the minimum and maximum of the weighted range across all the species considered

Symbol	Parameter definition	Default value	Range	Refs.
Bionomic parameters				
$\chi$	Human blood index	0.5	(0.01, 1)	
*M*	Parity proportion	0.6	(0.39, 0.79)	
$A_{0}$	Sac proportion	0.5	(0.16, 0.88)	
$\tau$	Resting period duration	3 days	(2, 6)	
Standard parameters				
$\theta _{d}$	Maximum time a mosquito unsuccessfully searches for a blood meal per day	0.33 days		[16]
$\theta _{s}$	Duration of the extrinsic incubation period	10 days		[16]
$P_{B}$	Probability that a mosquito bites after encountering a host	0.95		[16]
$P_{C}$	Probability that a mosquito finds a resting place after biting	0.95		[16]
$P_{D}$	Probability that a mosquito survives the resting phase	0.99		[16]
$P_{E}$	Probability that a mosquito lays eggs and returns to host-seeking	0.88		[16]
*N*	Total number of hosts	1000		
Intervention parameters				
$\beta _{r}$	Repelling effect		(0, 0.6)	[18]
$\beta _{d}$	Disarming effect		(0, 0.6)	[18]
$\beta _{m}$	Preprandial killing effect		(0, 0.6)	[18]
$\xi$	Postprandial killing effect		(0, 0.4)	[18]

## Results

Data used in the systematic review, from eligible studies, are provided in the Additional file 2: data file.

### Human blood index (HBI)

**Fig. 2:**
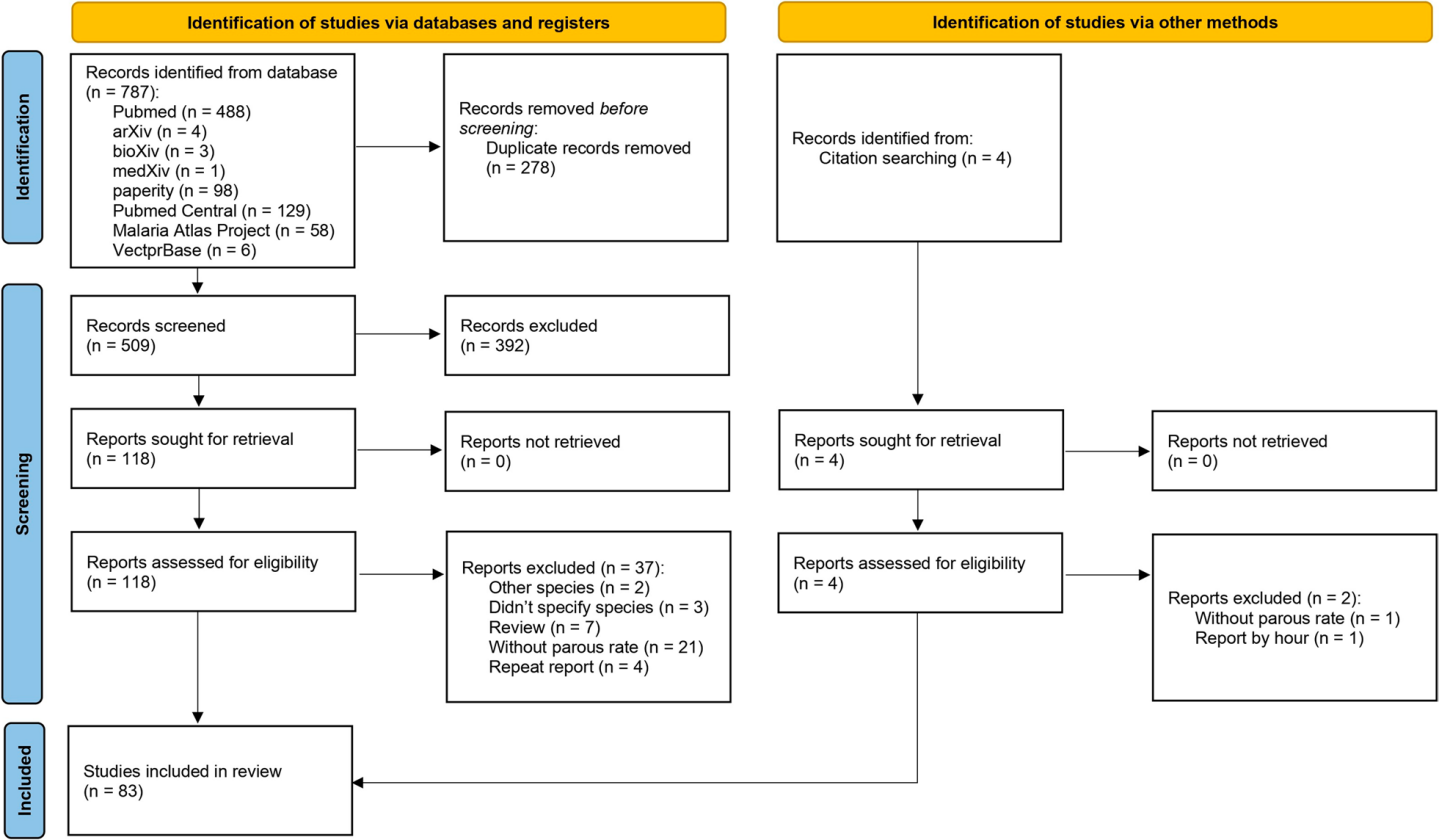
Preferred Reporting Items for Systematic Reviews and Meta-Analyses (PRISMA) flow diagram of HBI systematic search

A total of 128 publications were identified from databases and citation searches. After removing duplicate records, 90 abstracts of studies were screened, and 46 studies were considered relevant and assessed for eligibility following the inclusion and exclusion criteria. As a result, 39 studies with 102 data points were included in our final review (Fig. 2).

**Fig. 3:**
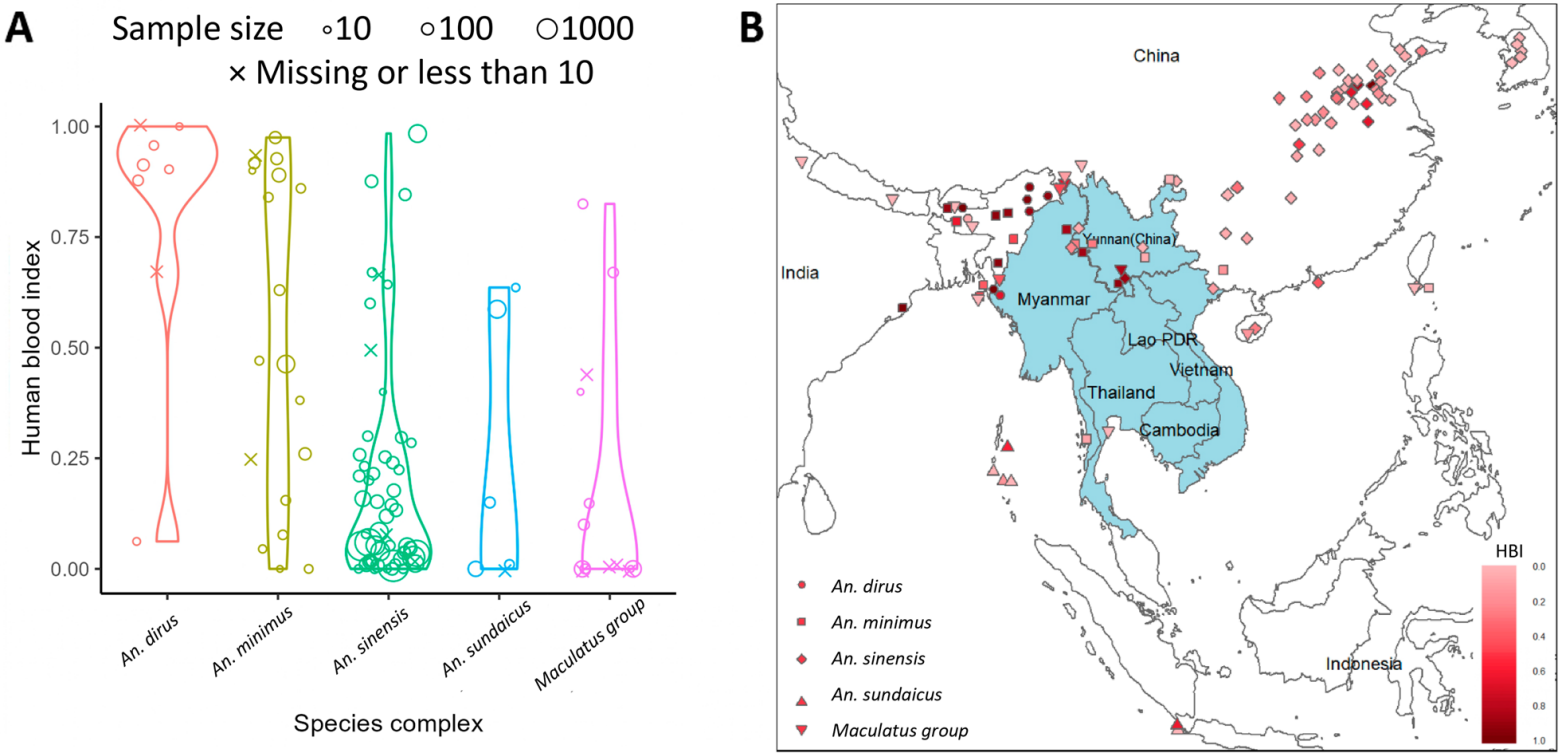
Overview of HBI **A** Distribution of HBI of different species complexes **B** Geographic distribution of study sites of HBI data points

The distribution for the HBI of these five species complexes can be compared in Fig. 3A. The weighted mean, range and 95% confidence quantile are provided in Table 4. Kruskal–Wallis test revealed a statistically significant difference in HBI between these species complexes (*P* < 0.01, effect size, 0.17). Dunn’s test indicated that *An. dirus* complex is significantly different from *An. sinensis* and Maculatus group; *An. minimus* complex is significantly different from *An. sinensis* (Bonferroni adjusted *P*-value < 0.05). *An. dirus* complex has a higher average HBI, while the average HBI of *An. sinensis* and the Maculatus group are lower. The HBI has a wide range and 95% confidence interval under different conditions. The geographical distribution of the data points is displayed in Fig. 3B. In addition, the distribution map for each species complex can be found in Additional file 3: Fig. S1.

Generally, host selection by mosquitoes can either be fixed (where host selection does not depend on the availability of the host) or opportunistic (where host selection depends on the availability of the host). For opportunistic mosquito species, the HBI can differ within a small geographical region, depending on the host availability or host accessibility [52]. Our review shows that *An. minimus* complex, *An. sinensis*, *An. maculatus* group and *An. sundaicus* complex display an opportunistic blood-feeding behaviour [25, 53–57], which means they are either anthropophilic or zoophilic, depending upon the availability of a local host. However, *An. dirus* complex is more anthropophilic [25, 55, 58].

### Parity proportion

**Fig. 4:**
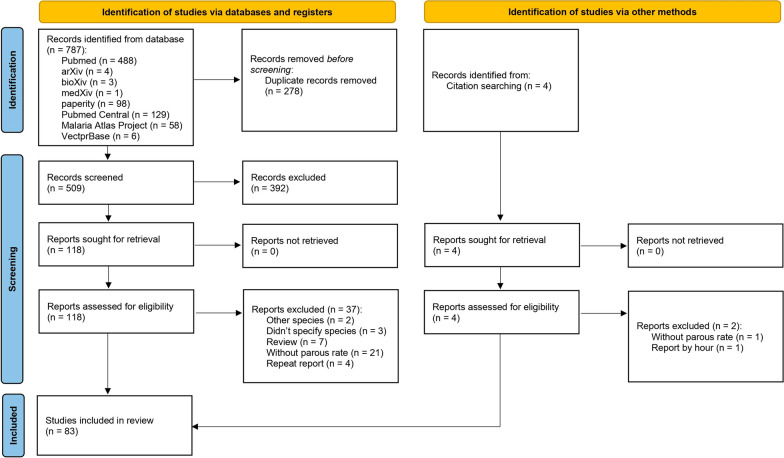
PRISMA flow diagram of parity proportion systematic search

On the basis of database searches and citation searches, 787 publications were identified. After eliminating duplicate records, abstracts from 509 studies were screened. The inclusion and exclusion criteria were then applied and 118 studies were found to be relevant. Finally, 83 studies comprising 266 data points were included in our final review (Fig. 4).

**Fig. 5:**
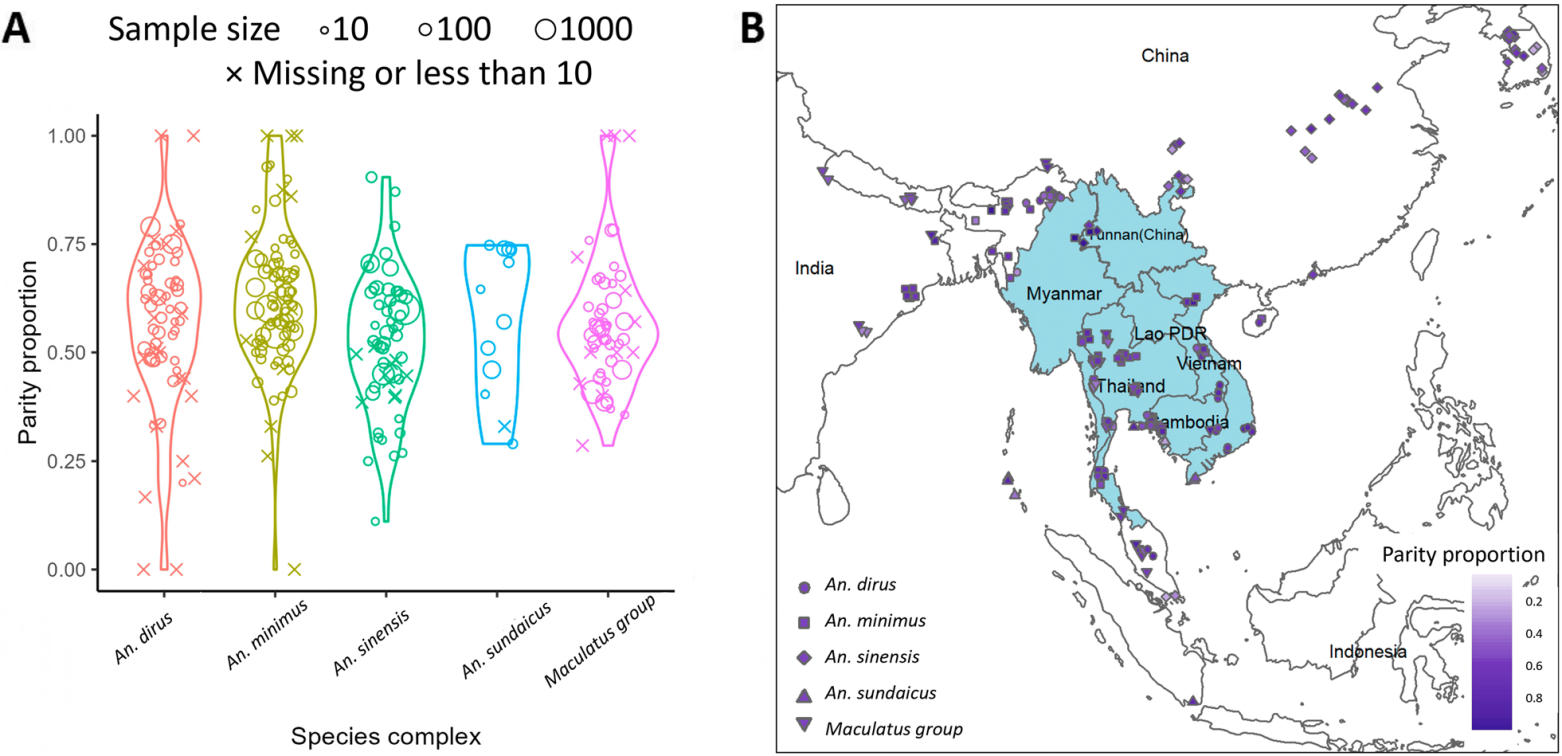
Overview of parity proportion **A** Distribution of parity proportion of different species complexes **B** Geographic distribution of study sites of parity proportion data points

Figure 5A shows the distribution of parity rates among these five species complexes. The weighted mean, range and 95% confidence quantile are provided in Table 4. The Kruskal–Wallis test revealed a statistically significant difference in parity proportion between these species complexes, while the effect size was small (*P* < 0.05, effect size, 0.04). However, Dunn’s test for multiply pairwise comparison did not indicate a statistically significant difference between groups after adjusting the *P*-value with Bonferroni correction. *An. dirus* complex has the highest average parity rate, while the average parity proportion of *An. maculatus* group is the lowest. The 95% confidence interval of the parity proportion among these species complexes is 0.39–0.79 under different conditions.

Figure 5B depicts the geographic distribution of the data points. In addition, the detailed distribution map for each species complex is provided in Additional file 3: Fig. S2.

The results of the univariate and multivariate logistic regression analyses are shown in Additional file 3: Table S3. Multivariate analysis indicated that using insecticide decreases the parity proportion. A lower parity proportion was also be found in the indoor collection versus other locations (outdoor, animal shelter, combined). The parity proportion using the resting collection was not significantly different from the whole night biting collection. In contrast, the half-night biting collection displayed a lower parity proportion, and the light trap collection indicated a higher parity proportion. Herbaceous habitat had a lower parity proportion compared with the forest, croplands and forest/cropland mosaics. The effect of climate zone on parity proportion differed for different species complex. *An. dirus* complex had a higher parity proportion in tropical regions compared with temperate regions, with weighted means of 58% and 51%, respectively. However, for *An. minimus* complex and *An. sinensis*, the parity proportion was higher in temperate regions than tropical regions, with weighted means of 66% and 59% for *An. minimus* and 53% and 27% for *An. sinensis*, respectively. For *An. sinensis*, the only species found in the cold region, the parity proportion in the cold region was higher than in the temperate and tropical regions, with a weighted mean of 61%.

### Sac proportion

During database and citation searches, four publications were found containing data on the proportion of mosquitoes with uncontracted ovary sacs, which can be used to estimate the time until mosquitoes return to host-seeking after laying eggs. After excluding one paper of other species, three papers [24, 59, 60] containing 22 data points were included in our review.

Two studies explored the sac proportion of *An. sinensis* collected by human/cow bait during the evening, midnight and before dawn. High sac proportions in these two papers indicated that most of the mosquitoes return to blood-seeking stage during that night after oviposition. However, the sac stage composition after midnight differs from these two papers. One contains more long sac types (stage AB 91.4%, stage CD 2.9%) [60], while the other one contains more noticeably contracted sac types (stage AB 35.4%, stage CD 43.1%) [59]. In the Cambodian study, sac proportion was 0.2 [24], which is much lower than results reported from China [59, 60].

**Fig. 6:**
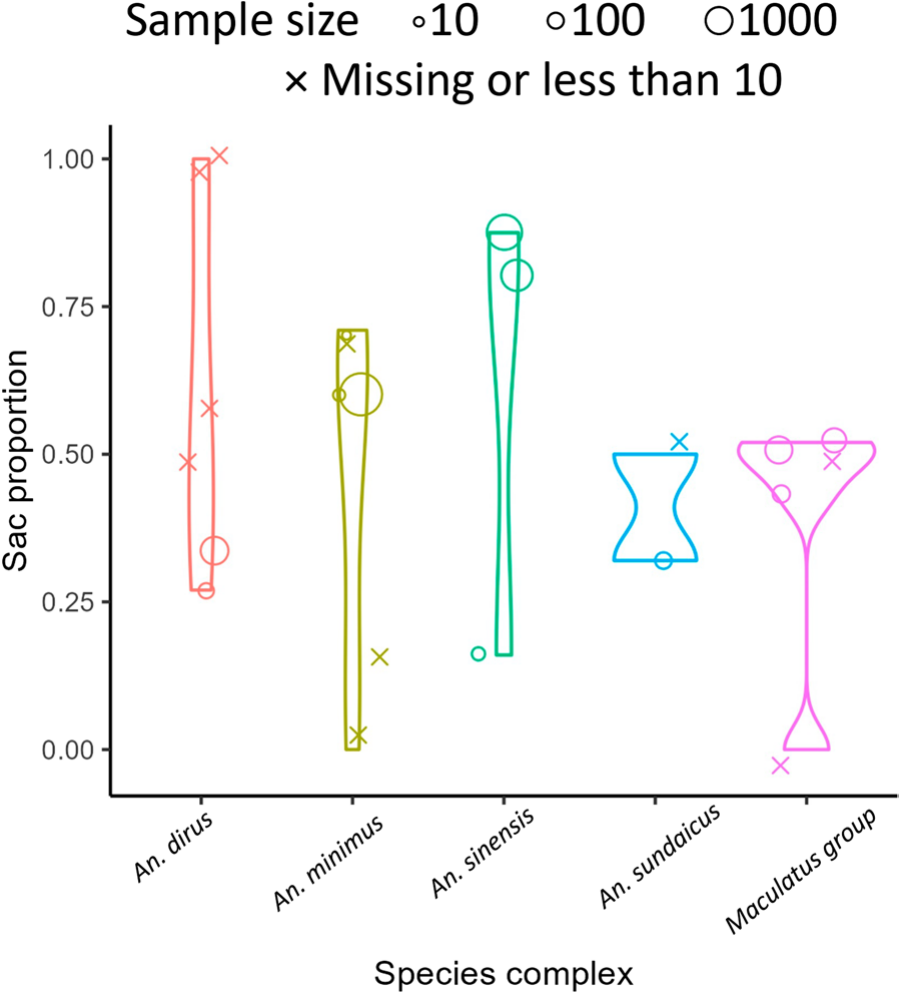
Distribution of sac proportion of different species complexes

The distribution of sac proportions among these five species complexes is shown in Fig. 6. The descriptive statistical information of sac proportion can be found in Table 4.

Due to having less than five samples in each group of species complex, we did not conduct a Kruskal–Wallis test.

### Resting period duration

We found 12 publications containing 18 data points of resting period duration for three species complexes: *An. minimus* complex, *An. sinensis* and *An. sundaicus* complex. The parameter ranges are listed in Table 4.

For *An. minimus* complex, field or laboratory observation indicated a 2–$-$2.5-day resting duration during the rainy season in India, Thailand and China [61–64]. Meanwhile, a 3–6-day duration during the dry, cool season was observed in Thailand and India [61, 63]. A study from Bangladesh illustrated a 2-day gonotrophic cycle from the evidence of 52% half gravid and 48% gravid mosquitoes from a 1-year collection [65]. For *An. sinensis*, the range of duration is 2–4 days in the rainy season in China or South Korea, measured by collecting the fully fed mosquito and observing until oviposition in the laboratory or natural conditions [60, 66–68]. The experiment with ambient temperature and relative humidity detected a maximum of 2.7 days in 23–25^∘^C, 89% relative humidity setting, and a minimum of 1.8 days in 31–35^∘^C, 88–$-$89.5% relative humidity setting [69]. The resting period duration of *An. maculatus* complex in the rainy season is 2–3 days in Malaysia, measured by capture–recapture studies [70, 71].

**Table 4:** Weighted mean, range and 95% confidence interval (CI) for each species complex and entomological parameter

Species	Number of sites$\dag$	Weighted mean$\dag$	Range$\dag$	Weighted 95% CI$\dag$
Human blood index (HBI)				
*An. dirus* (a)*	6	0.87	(0.06, 1)	(0.06, 1)
*An. minimus* (ab)	17	0.61	(0, 0.98)	(0.05, 0.98)
*An. sinensis* (c)	51	0.10	(0, 0.98)	(0.01, 0.98)
*An. sundaicus* (a)	5	0.34	(0, 0.64)	(0, 0.59)
Maculatus group (b)	9	0.09	(0, 0.83)	(0, 0.83)
Parity proportion				
*An. dirus* (a)	45	0.64	(0.20, 0.80)	(0.43, 0.79)
*An. minimus* (a)	68	0.60	(0.39, 0.93)	(0.48, 0.72)
*An. sinensis* (a)	48	0.57	(0.11, 0.90)	(0.41, 0.73)
*An. sundaicus* (a)	11	0.59	(0.29, 0.75)	(0.46, 0.74)
Maculatus group (a)	41	0.51	(0.16, 0.78)	(0.39, 0.78)
Sac proportion				
*An. dirus*	2	0.33	(0.27, 0.34)	(0.27, 0.34)
*An. minimus*	3	0.60	(0.60, 0.70)	(0.60, 0.60)
*An. sinensis*	3	0.82	(0.16, 0.88)	(0.16, 0.88)
*An. sundaicus*	1	0.32	(0.32, 0.32)	(0.32, 0.32)
Maculatus group	3	0.50	(0.43, 0.52)	(0.43, 0.52)
Resting period duration				
*An. minimus*	8		(2, 6)	
*An. sinensis*	4		(1.8, 4.3)	
Maculatus group	4		(2.3, 2.4)	

*Species complexes sharing a common letter were not significantly different (Kruskal–Wallis test and Dunn’s post hoc test). $\dag$ Only data points with more than ten mosquito samples were included

### Sensitivity analysis

**Fig. 7:**
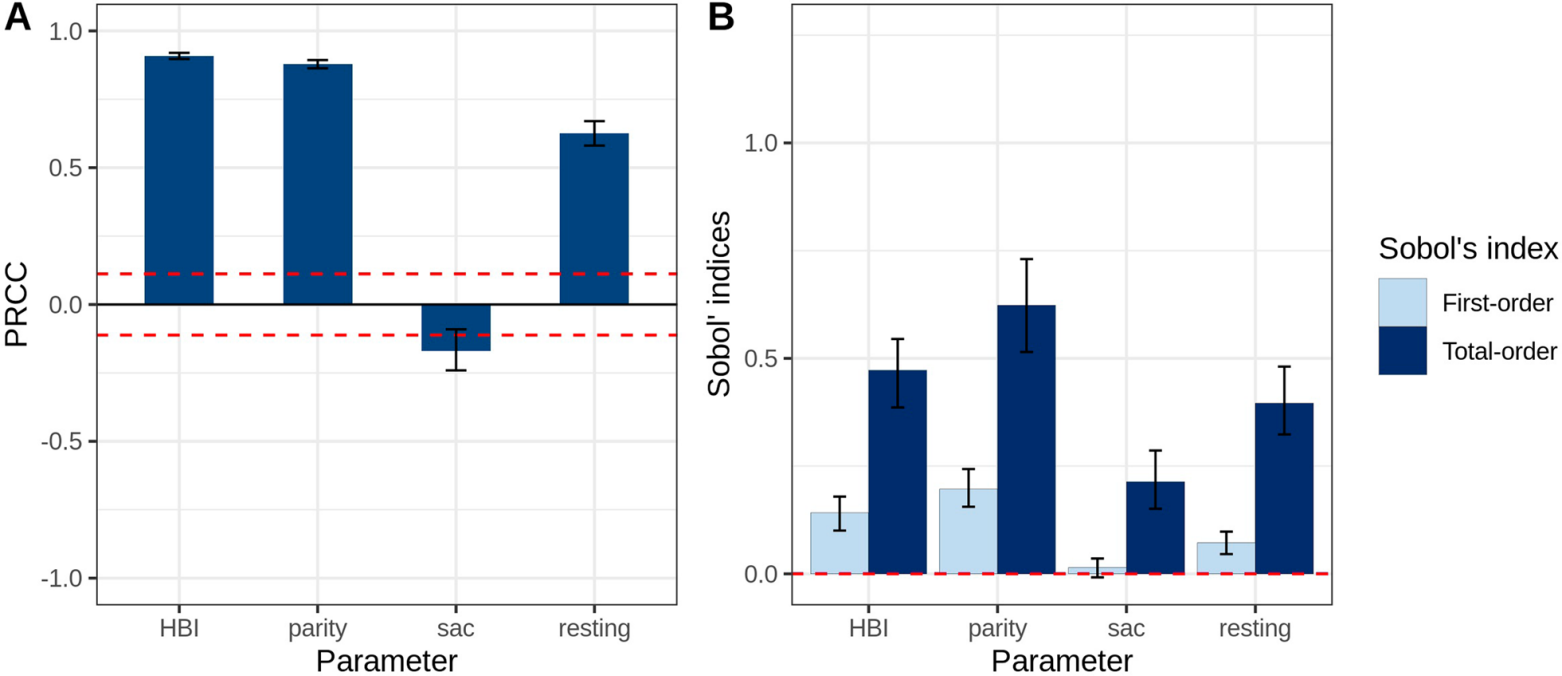
Global sensitivity analysis results for the bionomic parameters of the vectorial capacity model. **A** PRCC index. The area between the dashed lines represents PRCC values that are not statistically significant. **B** Sobol’s index. The area below the dashed lines represents Sobol’s indices that are not statistically significant

The sensitivity analysis results obtained from LHS-PRCC and Sobol’s method can be compared in Fig. 7A and Fig. 7B. LHS-PRCC and Sobol’s method revealed that the relative change of vectorial capacity was sensitive to all these bionomic parameters. It was most sensitive to HBI and parity proportion, while sac proportion was the least sensitive parameter. Further analysis of Sobol’s second order indices are listed in Additional file 3: Table S2, which indicates that larger values of vectorial capacity are associated with larger values of both HBI and parity proportion.

**Fig. 8:**
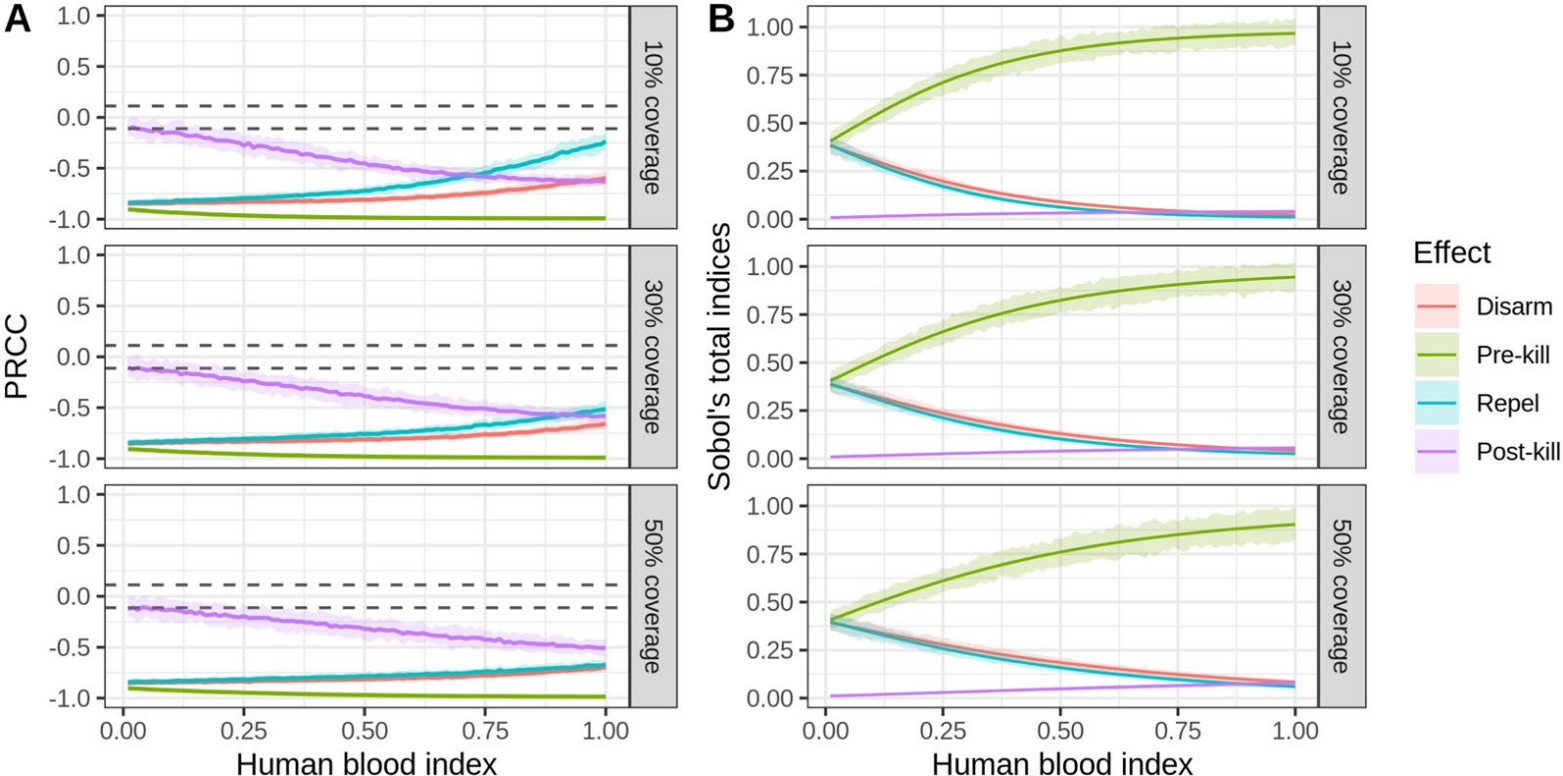
**A** The variation in sensitivity of the intervention parameters in different human blood index (HBI) settings under three intervention coverage levels, reported by PRCC. The area between the dashed lines represents PRCC values that are not statistically significant. **B** The variation in sensitivity of the intervention parameters for different HBI settings, reported by Sobol’s total index

**Fig. 9:**
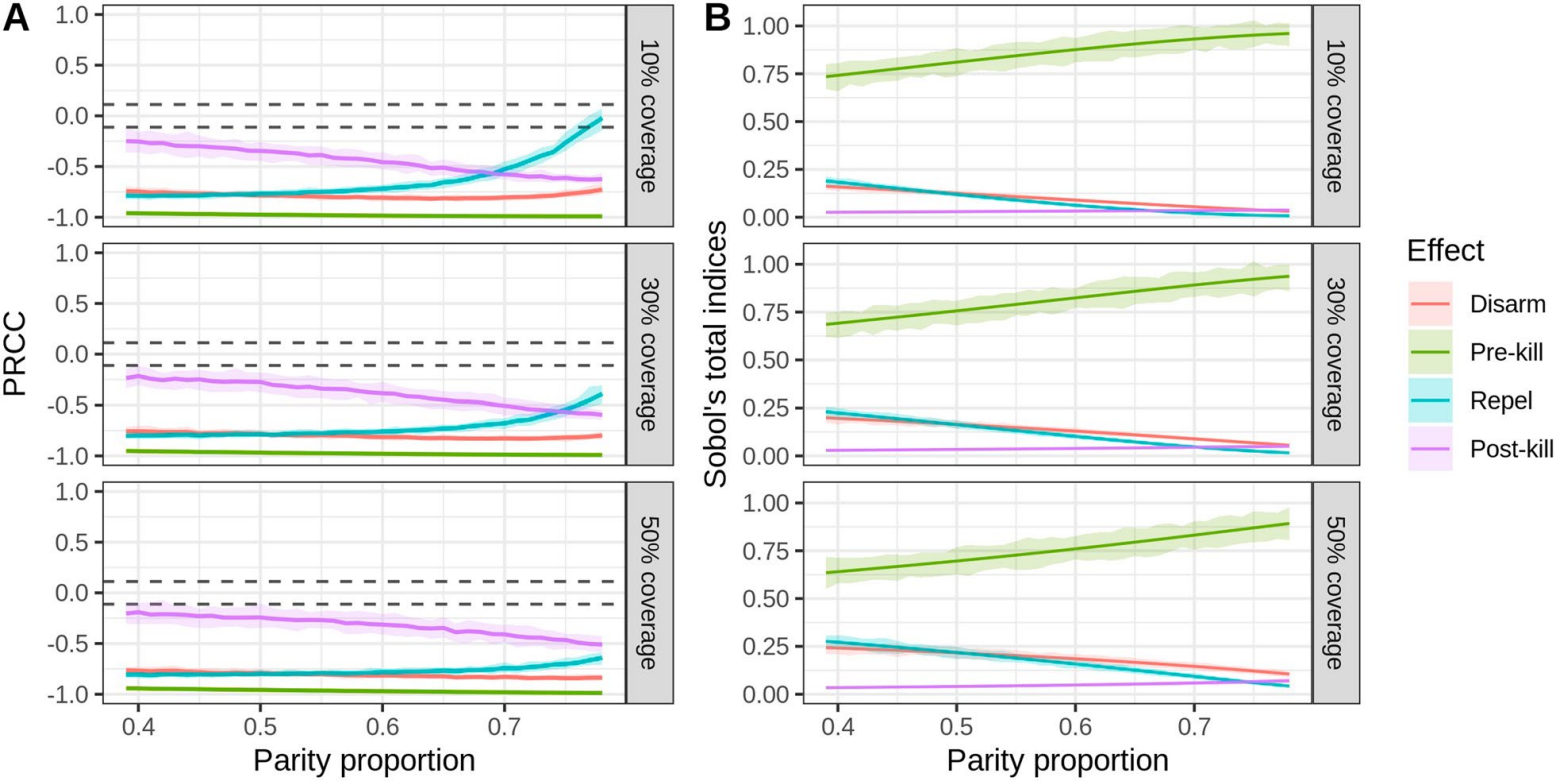
**A** The variation in sensitivity of the intervention parameters in different parity proportion settings under three intervention coverage levels, reported by PRCC. The area between the dashed lines represents PRCC values that are not statistically significant. **B** The variation in sensitivity of the intervention parameters for different HBI settings, reported by Sobol’s total index

**Fig. 10:**
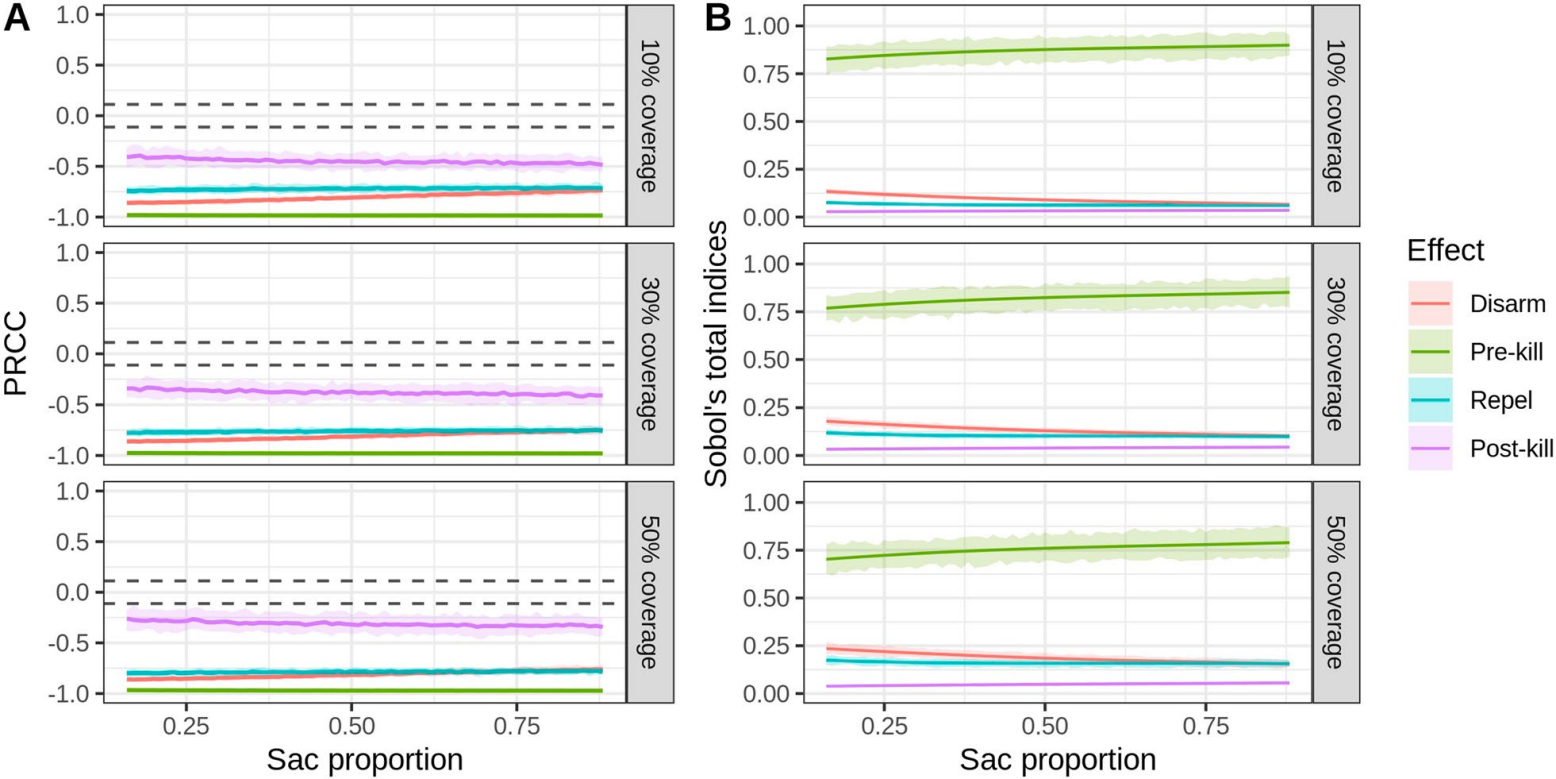
**A** The variation in sensitivity of the intervention parameters in different sac proportion settings under three intervention coverage levels, reported by PRCC. The area between the dashed lines represents PRCC values that are not statistically significant. **B** The variation in sensitivity of the intervention parameters for different HBI settings, reported by Sobol’s total index

The variation in the sensitivity index of the intervention parameters under different bionomic settings is shown in Fig. 8, 9 and 10. LHS-PRCC and Sobol’s total order index illustrated that, in a low HBI setting, a similar impact could be observed between preprandial killing, disarming and repelling effect, and all of them were more influential than the postprandial killing effect. However, preprandial killing had the most substantial impact in a high HBI/parity proportion setting. Comparing three different levels of coverage, we observed that disarming and repelling were more influential than the postprandial killing effect under high coverage level. In contrast, postprandial killing was more sensitive than repelling in low coverage settings when HBI/parity proportions were high. It is also worth noting that limited variation was observed under different sac proportion settings.

## Discussion

In this study, we performed a systematic literature search for key bionomics parameters to understand the life history characteristics of the main malaria vectors in the GMS. After generating a bionomics dataset of local species, we conducted global sensitivity analyses. This allowed us to identify the most influential mosquito bionomics, as well as the most effective modes of action of potential vector control tools, for reducing vectorial capacity.

Our systematic review provides detailed information regarding the bionomics of major malaria vector species in GMS. We found that bionomics differ from area to area, and several vital factors influence these bionomics. It is possible that these factors influence the species of the complex present. Previous studies demonstrated that the HBI could be affected by local human/animal ratio, host availability (e.g. using IRS, personal protection), trapping location (e.g. indoor or outdoor), trapping method, seasonality and homogeneity of mosquitoes [53, 54, 56, 72–75]. Several studies have shown that parity proportion is affected by geographical factors (land use type), climate and environmental factors (rainfall, temperature) and sampling factors (method, timing and location) [60, 68, 73, 74, 76–83]. Environmental factors also affect the resting period duration. The resting period duration increases when temperature or humidity decreases [69, 84–86], or when the mosquito activity is curtailed due to the rain [71]. Despite this, little information is available regarding sac proportion. Future investigations are needed to fill these knowledge gaps. A better understanding of these bionomics and vectorial capacity could support the development of more effective malaria control strategies in the GMS.

Compared with other vectorial capacity models derived from the Garrett–Jones equation, the Chitnis et al. [22] model can better integrate vector control intervention targeting different stages of the mosquito feeding cycle. It also considers the variation in the duration of the host-seeking stage and transmission delay caused by the resting period duration. Furthermore, using relative vectorial capacity as model output allowed us to compare the intervention effects in different environments. However, additional uncertainty arises due to the simple model assumptions: it assumes a constant mortality rate during each cycle and assumes mosquitoes feed only once each cycle. Previous vectorial capacity modelling studies have been summarised by Catano-Lopez et al. [21]. These studies have taken into account some of these factors: age-dependent vector mortality [87–89] or temperature related transmission parameters [90–93].

Another possible area of future modelling research would be to incorporate zoonotic malaria. According to a recent report, zoonotic malaria infections, caused by *P. knowlesi* and other simian *Plasmodium* species, mainly transmitted by vectors in forests and forest fringes, are on the rise in many Southeast Asian countries, threatening malaria elimination [94, 95]. To develop a full picture of malaria transmission in GMS, additional studies are needed.

Sensitivity analysis of vectorial capacity or reproductive number helps us to identify the most influential parameter regarding vector control. Most studies used differentiation-based local sensitivity analysis [96], assessing the parameter sensitivity around one particular point in the model input space [14, 97–99]. Considering the uncertainty regarding parameter values, we used global sensitivity analysis, which allowed us to explore a multi-dimensional input space and take into account the non-linearities and interactions in models [47]. Meanwhile, most research investigated the most influential biological parameter on the vectorial capacity or reproductive number, such as mosquito biting rate [97, 98], mosquito mortality[14] or mosquito–human contact rate [99]. However, some interventions could target more than one parameter, and different interventions could target the same parameter. With our modelling framework, we can quantitatively compare the intervention parameters and identify the most significant ones in different settings.

Malaria control programmes tend to take a one-fits-all approach [100, 101]. However, we show here that in different bionomic settings, different tools may be appropriate. In general, preprandial killing and disarming have a higher community-level effect on transmission blocking than repelling, since repelling could increase the risk for the non-intervention population, especially under low coverage levels. Previous research has shown that as some tools age, they lose preprandial killing efficacy, however, these mosquitoes are disarmed instead [18]. Our results suggest that in areas with low HBI, vector tools that focus on disarming, repelling or killing mosquitoes before they bite have a similar impact, suggesting that tools switching from preprandial mortality to disarming over time may maintain high impact. However, as with HBI and parity proportion, the impact of disarming and repelling decrease, especially at low intervention coverage, and therefore, programmes should focus on replacing tools more frequently to ensure mosquitoes are killed before they feed. For locations where the main vectors have fixed blood-feeding behaviour, we would expect similar HBIs, and therefore similar tools can be used. However, for locations with opportunistic vectors, a greater understanding of the local HBI (due to the availability of other non-human hosts) is needed to decide the modes of action required for efficient tools. Characteristics of tools explored in this study may come at different price points, with larger dosages of insecticide often associated with more preprandial killing [42]. This study highlights that these more expensive products may not be required in some bionomic settings.

## Conclusions

This study reviewed in detail the available information on HBI, parity proportion, sac proportion and the resting period duration in the GMS. Using these broad ranges of bionomic parameters, we performed a global sensitivity analysis of the vectorial capacity model.

Our intervention model, based on [17, 97], offered a framework to assess the community-level impact of different characteristics of vector control tools. Although preprandial killing is always the most desirable characteristic, we show that in different bionomic settings other modes of action may also be effective, potentially prolonging the time before a tool needs to be replaced. This could lead to more cost-effective, targeted malaria control.

### Supplementary Information

**Additional file 1.Additional information**, Equations for the interventions model; **Table S1**, Detailedparameter definitions, default values and range for the vectorial capacity model and intervention model. **FigureS1**, Geographic distribution of human blood index data points for different species complexes. **FigureS2**, Geographic distribution of parity proportion data points for different species complexes. **Table S2**, Parityproportion univariate and multivariate regression results. **Table S3**, Sobol's second order index results for theinfluence of human blood index, parity proportion, sac proportion and resting duration.

**Additional file 2.** Data extracted during systematic reviews.

**Additional file 3:  Table S1.** Detailed parameter definition, default value and range of vectorial capacity model and intervention model.** Figure S1.** Geographic distribution of HBI data points for different species complexes.** Figure S2.** Geographic distribution of parity proportion data points for different speciescomplexes.**Table S2.** Sobol’s second order index.**Table S3.** Univariate and multivariate regression results for parity proportion.

## Data Availability

Data collected in the systematic search and R scripts of the analysis are attached as Additional file 2.
