# Supplementary material for: Optimizing malaria vector control in the Greater Mekong Subregion: a systematic review and mathematical modelling study to identify desirable intervention characteristics
Source: Parasit Vectors. 2024 Mar 29;17:162. doi: 10.1186/s13071-024-06234-4 (PMC10981350; doi:10.1186/s13071-024-06234-4)
Supplement: Supplementary file 3 — Additional file 3: Table S1. Detailed parameter definition, default value and range of vectorial capacity model and intervention model. Figure S1. Geographic distribution of HBI data points for different species complexes. Figure S2. Geographic distribution of parity proportion data points for different speciescomplexes.Table S2. Sobol’s second order index.Table S3. Univariate and multivariate regression results for parity proportion. [file 13071_2024_6234_MOESM3_ESM.pdf]

# Optimizing malaria vector control in the Greater Mekong Subregion: A systematic review and mathematical modelling study to identify desirable intervention characteristics

Yuqian Wang[1,2], Nakul Chitnis[1,2], and Emma L Fairbanks[1,2]

[1]Department of Epidemiology and Public Health, Swiss Tropical and Public Health Institute, Kreuzstrasse 2, 4123 Allschwill, Basel, Switzerland
[2]University of Basel, Petersplatz 1, 4001, Basel, Switzerland
[*]Corresponding author: emma.fairbanks@unibas.ch

# 1 Equations in the intervention model

We use an *Anopheles* feeding cycle model first described in Chitnis et al. [1] and Briët et al. [2]. The framework for intervention effects has previously been utilized and described in Denz et al. [3] and Fairbanks et al. [4]. Here we provide an overview of these frameworks. A detailed description of parameters are listed in Table S1.

Vectorial capacity are calculated as

$$\Psi_{ij} = \overbrace{\frac{N_{v0}}{1-P_A-P_{df}}}^{\Psi_{ij}^{(A)}} \times \overbrace{\frac{P_{A^j}P_{B_j}P_{C_j}P_{D_j}P_{E_j}}{N_j}}^{\Psi_{ij}^{(B)}} \times \overbrace{\left[\left(\sum_{h=0}^{k_+}\binom{\theta_s-(h+1)\tau+h}{h}P_A^{\theta_s-(h+1)\tau}P_{df}^h\right)\right.}^{\Psi_{ij}^{(C)}\ldots}$$
$$\underbrace{\left.+\sum_{l=1}^{\tau-1}\left(\sum_{h=0}^{k_{l+}}\binom{(\theta_s+l)-(h+2)\tau+h}{h}P_A^{(\theta_s+l)-(h+2)\tau}P_{df}^{h+1}\right)\right]}_{\ldots\Psi_{ij}^{(C)}} \times \underbrace{\frac{P_{A^i}P_{B_i}}{1-P_A-P_{df}}}_{\Psi_{ij}^{(D)}}. \tag{1}$$

In the vectorial capacity equation, $\Psi_{ij}^{(A)}$ represents the number of mosquitoes seeking for a host in a single day, $\Psi_{ij}^{(B)}$ indicates the probability of mosquitoes bite a type j host during that day and live through a feeding cycle, $\Psi_{ij}^{(C)}$ is the probability of mosquitoes survive enough time to become infective, and the total number of potential bite on type i host is calculated as $\Psi_{ij}^{(D)}$ [1].

The host availability rates are calculated as

$$\alpha_i = \frac{1}{N_i}\left(\frac{P_{A^i}}{1-P_A}\right)\left(\frac{-\ln P_A}{\theta_d}\right). \tag{2}$$

Derivation of host availability rate can be found in Additional file 2 in Briët et al. [2].

Then we can consider the intervention effects as

**Host availability rate of protected hosts:**

$$(1-min(\beta_r+\beta_m+\beta_d,1))\times\alpha_{Hb},$$

where $\alpha_{Hb}$ is the availability rate of a human host not protected by the intervention, $\beta_r$ is the repelling effect, $\beta_m$ preprandial killing effect, and $\beta_d$ is the disarming effect. The reduction in biting is set to a maximum of 1, since the host availability rate is larger than or equal to 0.

**Probability of surviving to lay eggs after biting a protected human host:**

$$(1 - \xi) \times P_c,$$

where $P_c$ is the probability for a mosquito that finds a resting place after biting a unprotected human host and $\xi$ is the probability a mosquitoes, which would have survived biting a human host without the intervention, dies postprandially if they bite a protected host.

**Rate of disarming or preprandial killing:** This is modelled as a dummy host, which does not contribute to malaria transmission, with an availability rate of

$$(\beta_m + \beta_d) \times \alpha_{Hb}. \tag{3}$$

**Probability of surviving to lay eggs, given the mosquito is preprandially killed or disarmed:**

$$\left(\frac{\beta_d}{\beta_d + \beta_m}\right).$$

# References


[1] N Chitnis, T Smith, and R Steketee. A mathematical model for the dynamics of malaria in mosquitoes feeding on a heterogeneous host population. *Journal of Biological Dynamics*, 2(3):259–285, 2008. doi: 10.1080/17513750701769857.

[2] OJT Briët, DE Impoinvil, N Chitnis, E Pothin, JF Lemoine, J Frederic, and TA Smith. Models of effectiveness of interventions against malaria transmitted by *Anopheles albimanus*. *Malaria journal*, 18(1):263, 2019. doi: 10.1186/s12936-019-2899-3.

[3] A Denz, MM Njoroge, MM Tambwe, C Champagne, F Okumu, JJA van Loon, A Hiscox, A Saddler, U Fillinger, SJ Moore, and N Chitnis. Predicting the impact of outdoor vector control interventions on malaria transmission intensity from semi-field studies. *Parasites & Vectors*, 14(1):64, 2021. doi: 10.1186/s13071-020-04560-x.

[4] EL Fairbanks, M Saeung, A Pongsiri, E Vajda, Y Wang, DJ McIver, JH Richardson, A Tatarsky, NF Lobo, SJ Moore, A Ponlawat, T Chareonviriyaphap, A Ross, and N Chitnis. Inference for entomological semi-field experiments: Fitting a mathematical model assessing personal and community protection of vector-control interventions. *Comput Biol Med*, 168:107716, 2024. doi: 10.1016/j.compbiomed.2023.107716.

Table S1: Detailed parameter definition, default value and range of vectorial capacity model and intervention model.

| Symbol | Parameter definition | Default value | Range | Ref. |
|---|---|---|---|---|
| | Bionomic parameters | | | |
| $\chi$ | Human blood index | 0.5 | (0.01, 1) | |
| $M$ | Parity proportion | 0.5 | (0.39, 0.78) | |
| $A_0$ | Sac proportion | 0.5 | (0.16, 0.88) | |
| $\tau$ | Resting period duration | 3 days | (2, 6) | |
| | Standard parameters | | | |
| $\theta_d$ | Maximum time a mosquito unsuccessfully searches for a blood meal per day | 0.33 days | | [2] |
| $\theta_s$ | Duration of the extrinsic incubation period | 10 days | | [2] |
| $P_B$ | Probability that a mosquito bites after encountering a host | 0.95 | | [2] |
| $P_C$ | Probability that a mosquito finds a resting place after biting | 0.95 | | [2] |
| $P_D$ | Probability that a mosquito survives the resting phase | 0.99 | | [2] |
| $P_E$ | Probability that a mosquito lays eggs and returns to host-seeking | 0.88 | | [2] |
| $N$ | Total number of hosts | 1000 | | |
| $N_{v0}$ | Total number of emerging mosquitoes that survive to seek for blood meal each day | | | |
| | Derived parameters | | | |
| $P_A$ | Probability of a mosquito that does not find a host or die after searching for blood meal for one night | | | |
| $P_{A^i}$ | Probability of a mosquito finds a type i host during one night blood meal search | | | |
| $P_{df}$ | Probability of a mosquito encounters a host and live through the whole feeding cycle | | | |
| $P_f$ | Probability of a mosquito lives through a feeding cycle | | | |
| | Intervention parameters | | | |
| $\beta_r$ | Repelling effect | | (0, 0.6) | [4] |
| $\beta_d$ | Disarming effect | | (0, 0.6) | [4] |
| $\beta_m$ | Preprandal killing effect | | (0, 0.6) | [4] |
| $\xi$ | Postprandal killing effect | | (0, 0.4) | [4] |

## 2 Geographical distribution figures

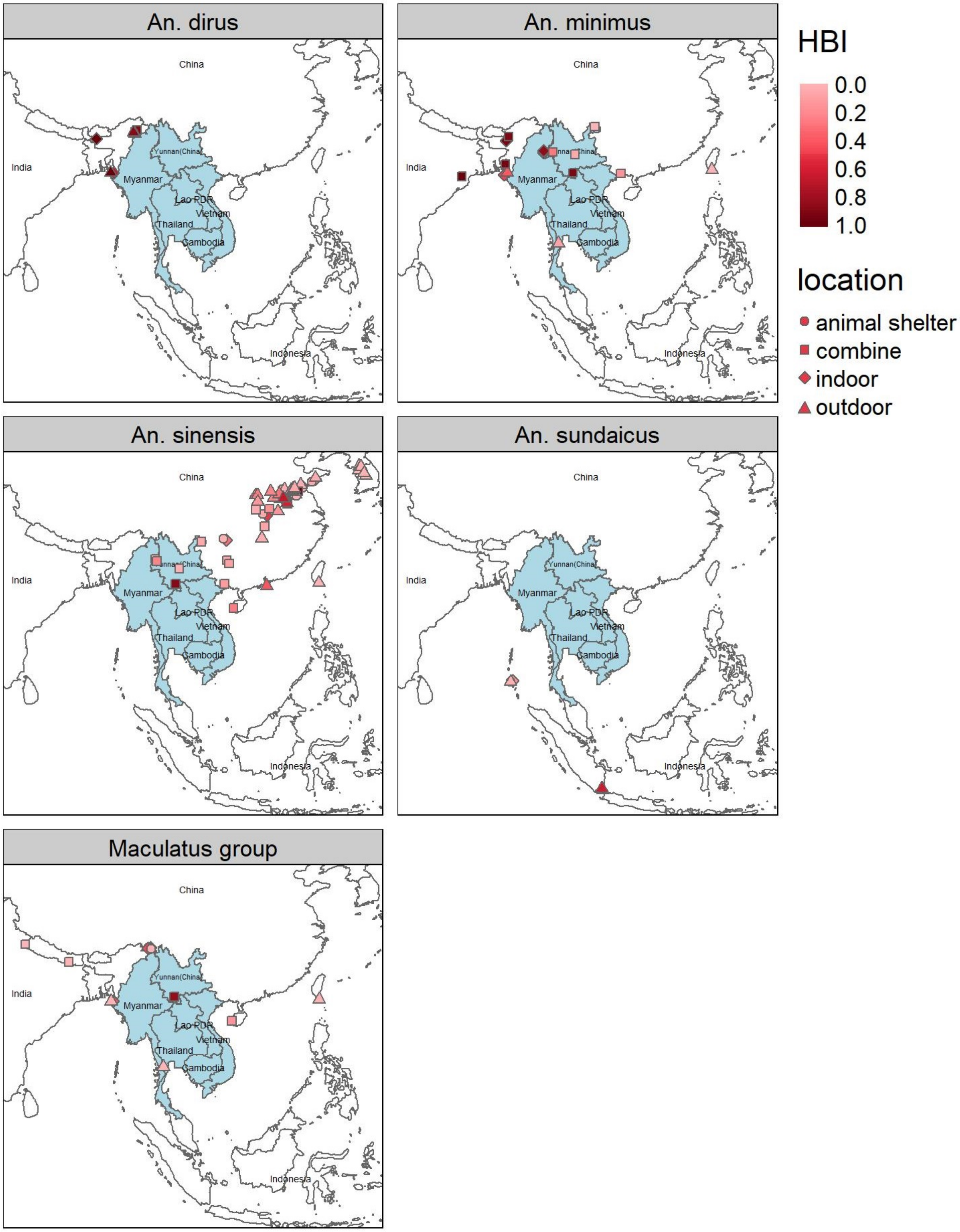


Figure S1: Geographic distribution of HBI data points for different species complexes

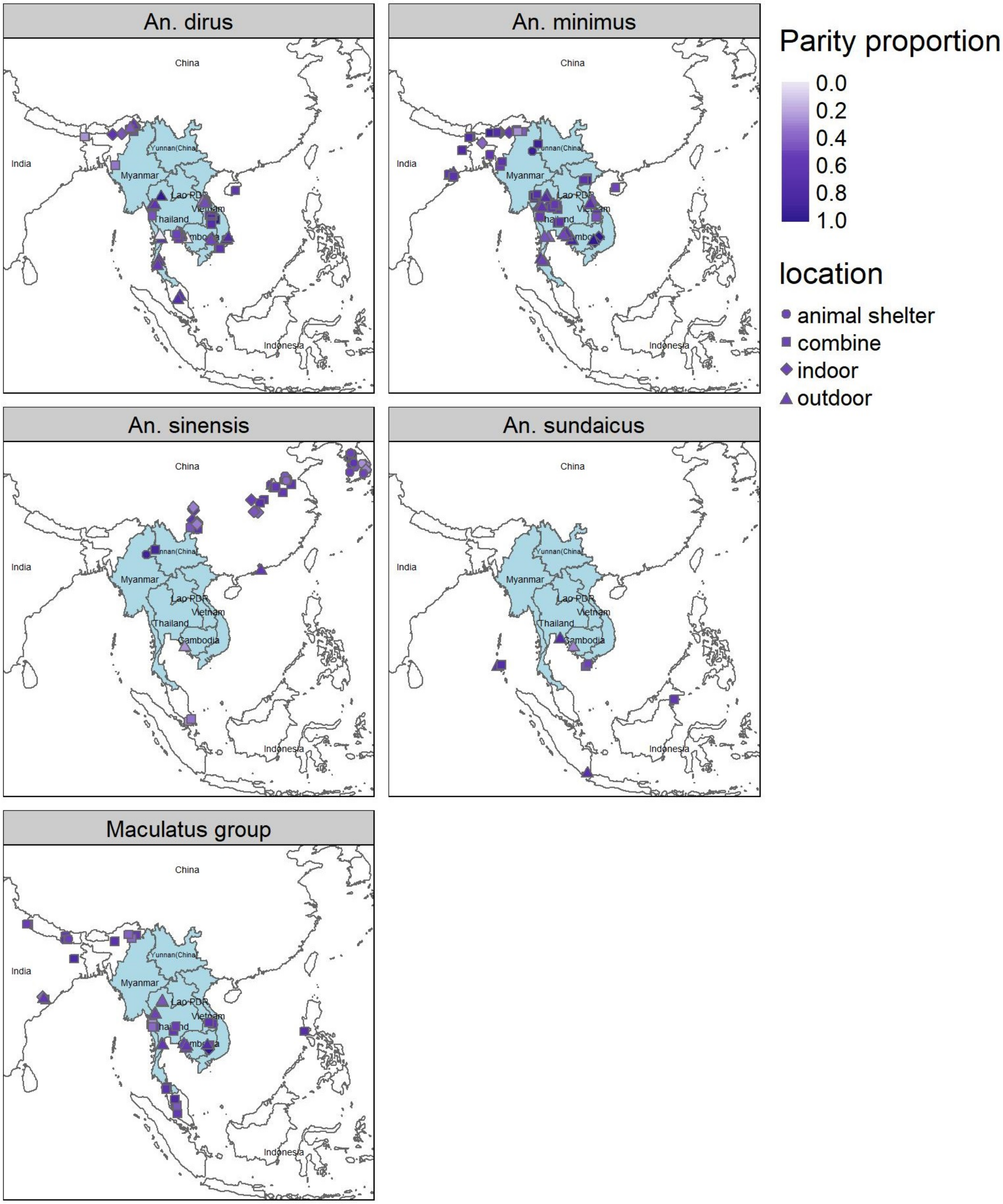


Figure S2: Geographic distribution of parity proportion data points for different species complexes

# 3 Parity rate logistic regression results

Table S2: Univariate and multivariate regression results for parity proportion.

| Variable | | OR (univariate) | OR (multivariate) |
|---|---|---|---|
| Species complex | Maculatus group | - | - |
| | *An. dirus* | 1.72 (1.65–1.79, p<0.001) | 1.18 (1.06–1.30, p=0.002) |
| | *An. minimus* | 1.40 (1.36–1.45, p<0.001) | 1.91 (1.70–2.15, p<0.001) |
| | *An. sinensis* | 1.28 (1.24–1.32, p<0.001) | 1.10 (1.02–1.19, p=0.017) |
| | *An. sundaicus* | 1.37 (1.30–1.45, p<0.001) | 1.21 (1.12–1.32, p<0.001) |
| Insecticide | No | - | - |
| | Yes | 0.87 (0.85–0.89, p<0.001) | 0.82 (0.79–0.85, p<0.001) |
| Season | Dry | - | - |
| | Rainy | 0.86 (0.83–0.89, p<0.001) | 0.99 (0.94–1.03, p=0.509) |
| | Both | 0.80 (0.77–0.83, p<0.001) | 0.92 (0.87–0.96, p<0.001) |
| Location | Indoor | - | - |
| | Animal shelter | 1.48 (1.39–1.58, p<0.001) | 1.33 (1.20–1.46, p<0.001) |
| | Combined | 1.09 (1.05–1.14, p<0.001) | 1.10 (1.04–1.17, p<0.001) |
| | Outdoor | 1.48 (1.42–1.55, p<0.001) | 1.24 (1.15–1.32, p<0.001) |
| Method | Biting (whole night) | - | - |
| | Biting (half night) | 1.13 (1.07–1.19, p<0.001) | 0.82(0.77–0.88, p<0.001) |
| | Combined | 0.98 (0.95–1.00, p=0.079) | 1.20 (1.13–1.26, p<0.001) |
| | Light trap | 1.30 (1.24–1.37, p<0.001) | 1.18 (1.08–1.28, p<0.001) |
| | Odour trap | 1.03 (0.94–1.13, p=0.552) | 0.48 (0.17–1.33, p=0.158) |
| | Resting | 0.92 (0.88–0.96, p<0.001) | 1.00 (0.94–1.07, p=0.958) |
| Land use | Herb | - | - |
| | Cropland | 0.82 (0.79–0.85, p<0.001) | 1.18 (1.09–1.27, p<0.001) |
| | Forest | 0.81 (0.79–0.84, p<0.001) | 1.22 (1.14–1.31, p<0.001) |
| | Forest/cropland | 0.85 (0.81–0.89, p<0.001) | 1.17 (1.08–1.26, p<0.001) |
| | Urban | 0.71 (0.63–0.80, p<0.001) | 1.16 (0.90–1.49, p=0.258) |
| Climate | Temperate | - | - |
| | Cold | 1.33 (1.28–1.37, p<0.001) | 1.09 (1.03–1.16, p=0.005) |
| | Tropical | 1.21 (1.18–1.25, p<0.001) | 1.17 (1.08–1.27, p<0.001) |
| | *An. dirus*:tropical | | 1.57 (1.40–1.77, p<0.001) |
| | *An. minimus*:tropical | | 0.72 (0.64–0.81, p<0.001) |
| | *An. sinensis*:tropical | | 0.36 (0.25–0.51, p<0.001) |

# 4 Sensitivity analysis results

Table S3: Sobol's second order index

| Parameters | Index (95% confidence interval) |
|---|---|
| HBI . parity | 0.14 (0.10–0.18) |
| HBI . sac | 0.00 (-0.01–0.01) |
| HBI . resting duration | 0.06 (0.04–0.09) |
| Parity . sac | 0.05 (-0.02–0.14) |
| Parity . resting duration | 0.09 (0.05–0.13) |
| Sac . resting duration | 0.05 (0.02–0.07) |
